# Recent advances in nanotechnology for eradicating bacterial biofilm

**DOI:** 10.7150/thno.67296

**Published:** 2022-02-28

**Authors:** Célia Sahli, Sergio E. Moya, John S. Lomas, Christine Gravier-Pelletier, Romain Briandet, Miryana Hémadi

**Affiliations:** 1 Université de Paris, CNRS, ITODYS, F-75013 Paris, France.; 2 Centro de Investigación Cooperativa en Biomateriales (CIC BiomaGUNE), 20009 Donostia-San Sebastián, Guipúzcoa, Spain.; 3 Université de Paris, CNRS, Laboratoire de Chimie et Biochimie Pharmacologiques et Toxicologiques, F-75006 Paris, France.; 4 Université Paris-Saclay, INRAE, AgroParisTech, Micalis Institute, Jouy-en-Josas, France.

**Keywords:** biofilms, bacteria, antibiotics, antimicrobials, nanotechnology, nanoparticles, therapy, diagnosis.

## Abstract

Microorganisms grouped together into spatially-organized communities called biofilms, are the cause of dramatic chronic infections in plants, animals and humans. In this review, the characteristics of biofilms and their interactions with antimicrobials are first described. Limitations of antibiotic treatments are discussed, and state-of-the-art alternative approaches based on the use of polymer, lipid, organic, inorganic and hybrid nanoparticles are presented, highlighting recent achievements in the application of nanomaterials to the field of theranostics for the eradication of biofilm. The aim of this review is to present a complete vision of nanobiotechnology-based approaches for eradicating bacterial biofilms and fighting antimicrobial tolerance.

## Introduction

The year 2020 was for the whole world the year of the Coronavirus, more precisely of the SARS-CoV-2 virus which causes an illness known as Covid-19. The on-going pandemic has highlighted the role of viruses as vectors of infectious diseases. The layman generally does not distinguish between a virus and a bacterium, even less the other classes of micro-organisms, but in the 1980s the general public, perhaps for the first time, learnt what a virus looks like, thanks to illustrations of the human immunodeficiency virus (HIV). The HIV is associated with sexual behaviour and intravenous drug use, whereas the latest coronavirus is for everybody. Other new viral diseases, notably SERS, MERS and Ebola, have tended to be geographically circumscribed, and the present coronavirus is by far the most “successful” in its ability to travel and to infect very large populations. However, this recent surge of viral diseases should not lead us to ignore the fact that many of the great pandemics, or plagues, of the past were caused by bacterial infections. Medieval plagues, such as the Black Death, which decimated populations over the greater part of Europe and Africa, were caused by a bacterium, *Yersinia pestis*. Cholera and typhus, which have been responsible for pandemics in recent years, are also bacterial diseases. Bacteria are everywhere, some benevolent, such as those in our digestive system, but many of them are responsible for chronic infections, and are becoming increasingly resistant to treatment. An imbalance in the microbiota can lead to many pathologies involving biofilms, the most important of which are listed in **Table 1**. Pathogenic bacteria lead to severe illnesses worldwide, thus increasing mortality in the short and long run.

Infections caused by bacteria highly resistant to antibiotics have been classified by the World Health Organization (WHO) among its top 10 research priorities. Globally, the number of deaths associated with antimicrobial resistance is estimated at 7 million per year. Mortality could rise to 10 million deaths per year in 2050 if effective therapies are not found 1. Besides mortality, the spread of bacterial diseases is expected to increase the costs of health care to more than 1.5 billion euros / year in Europe 2.

Selection pressure has led to the development of certain phenotypes of bacteria, the variations of which give an advantage in survival and reproduction. Only resistant bacteria survive, the others being killed by antibiotics. ESKAPE pathogens (*Enterococcus faecium*, *Staphylococcus aureus*, *Klebsiella pneumoniae*, *Acinetobacter baumannii*, *Pseudomonas aeruginosa*, and *Enterobacter* species) are bacteria capable of escaping most common antibiotics by the acquisition of antimicrobial-resistant genes 3. This resistance results from the misuse of antibiotics, in particular broad-spectrum antibiotics, in the treatment of human diseases as well as in the agricultural sector.

Bacterial antibiotic resistance is a scourge that appeared with the introduction of the first antibiotics. Many antibiotics discovered in the 1940s, such as penicillin, introduced in 1941, encountered resistance shortly after their distribution to the general public. Resistance appears earlier and earlier, notably because antibiotics in clinical use focus on a limited number of biological targets. There is now a race between the production of new antibiotics and the appearance of bacterial resistance.

In addition to this phenomenon, a very common strategy used by bacteria for their survival is grouping together into spatially organized communities called biofilms. Biofilms are involved in more than 65% of nosocomial infections 4, related to implanted equipment such as joint prostheses and heart valves 4, and also associated with chronic infections such as those involved in cystic fibrosis 5. Chronic wounds or ulcers have increased significantly during recent years in correlation with the increase of pathologies such as cancer, diabetes and obesity. Indeed, bacterial biofilm-associated infections are an important cause of death in victims of cystic fibrosis and viral infections. Thus, the complexity and severity of biofilm infections has urged researchers to study the molecular mechanisms involved in the formation and growth of biofilms, in order to identify the key steps that could be exploited to eradicate them.

Current control strategies for biofilms are dominated by chemical biocides and antiseptic solutions, which produce harmful by-products, and antibiotics with limited efficacy on resistant and slow-growing, persistent bacteria. Biofilms are hot-spots for the emergence of resistant mutants, genetic transfer of mobile elements and a source of antimicrobial tolerance. The advent of nanotechnology and especially nanomedicine has opened up new possibilities for diagnosis and treatment of many diseases. Due to their size and surface properties, nanomaterials have the potential to overcome physiological barriers. Nanoparticles (NPs) have been studied extensively in the last decade as drug carriers in many different fields, including oncology, immunotherapy, and neuroscience 6,7. They also offer many possibilities for eradicating bacteria, especially if combined with active molecules 8.

Scientific and medical studies on antibacterial treatments for biofilms constitute a major research field of the utmost importance. This research has led to many treatments that are expected to enhance the activity of currently used antibiotics or inhibitors either present in their free state or incorporated into a formulation. In this review, we first present the specificities of microorganisms living as biofilms, followed by an overview of the strategies used to treat bacterial biofilm communities and their drawbacks. Alternative therapies, effective ways of delivering antibiotics into the biofilm, and means for localized antibacterial action at a surface will be discussed. Finally, the contribution of nanotechnology, new advances in the field, and developments of theranostics will be described.

## 2. Microorganisms living within biofilms

This section constitutes an introduction to biofilms, which represent a common life-style for bacteria, one which gives them many advantages, particularly much higher tolerance to antimicrobial agents than free planktonic bacteria. We describe their life cycle, their architecture, how they function, and how the bacteria communicate and protect themselves from environmental stresses.

### 2.1 Formation and characteristics of microbial biofilms

Confronted by hostile environmental changes, the genetic richness of microorganisms allows them to survive and to multiply as well as to develop the host genome. There are between 15 000 and 30 000 different species of bacteria in the human body, about 38 trillion bacteria in a 70 kg "reference" man, between 20 and 30 years old 9. A large number of bacteria live on the skin, in the digestive tract as well as in the ear, nose and throat sphere. Most are either harmless or beneficial, when a balance between bacteria and host is established 10. The balance between pathogenic and beneficial bacteria in the intestine is a criterion determining good health and sickness. If an imbalance is present, called dysbiosis, the host-microorganism relationship is altered, and conditions such as inflammatory bowel disease, colon cancer, diabetes and obesity can emerge. The formation and accumulation of bacterial biofilms disrupts this balance and is one of the major causes of chronic infections 11.

Biofilms represent a much higher level of social and spatial organization than free single bacteria 5,12. The term “biofilm” refers to surface-associated microbial communities surrounded by a self-produced polymer matrix made of extracellular polymeric substances (EPS), typically composed of a mixture of exopolysaccharides, extracellular DNA (eDNA), and proteins 13. It is important to distinguish between sessile (adhering) bacteria and planktonic (free-swimming) organisms. Bacteria in biofilms are clearly different from their planktonic counterparts. The biofilm mode of growth is the predominant life-mode of most bacterial species. It is estimated that 40 to 80% of the microbial biomass on earth is associated with a biofilm 12. Microbial biofilms have been compared to human cities in which individuals choose a city, select the neighborhood that best suits their needs, set up their homes amongst others, and occasionally leave when life conditions deteriorate 14.

The matrix occupies most of the biofilm volume (typically 85%) and strengthens its structure while retaining great mechanical plasticity to the structure. Its composition depends on the microbial communities and the growth environment. These sessile biostructures are frequently vascularized by a network of water channels that allows nutrients as well as oxygen to be delivered to bacteria in the bulk, and to reject waste 15. Within biofilms, significant gradients appear: oxygen, pH or substrates. The biofilm spatial structure depends on these gradients which generate heterogeneous local microenvironments and diversification of cell types, including slow-growth and persister subpopulations which show a very high tolerance to disinfectants and antibiotics 16.

The biofilm way of life is adopted by bacteria in stressful situations as a form of protection. For the bacteria to survive and grow even under hostile conditions, the biofilm matrix adsorbs and retains nutrients and water 17. In order to cope with changing environmental conditions, bacteria adapt their metabolism, for example, by switching from respiration to fermentation if the amount of oxygen in the medium is limited 18.

Biofilms can form on any type of surface, biotic or abiotic. In humans, there are important sites for the formation of biofilms. Biofilms are often made up of several different bacterial species. This can result in a competition for nutrients and space, or in a synergy that allows the development of phenotypes beneficial for the survival and propagation of the biofilm. Bacteria communicate with each other through a phenomenon called quorum sensing (QS), which is an intercellular signaling pathway that allows bacteria to coordinate gene regulation and group activity in response to population density. It is a mode of communication between bacteria of the same species, but also of different species. This system is a key regulatory mechanism, based on the production of diffusible signaling molecules (autoinducers) which allow biofilms to adapt to changing circumstances 19. In general, Gram-negative bacteria use acyl-homoserine lactones (AHL) as autoinducers, and Gram-positive bacteria use processed oligo-peptides to communicate. In some cases, QS directs the production of the essential components of the biofilm such as biosurfactants or eDNA 20. Indeed, surfactants keep channels open by modifying cell-cell interactions and the attachment of bacteria to surfaces by adhesins. In *P. aeruginosa* bacteria, rhamnolipid biosurfactants are responsible for the complex 3D architecture of the biofilm 21.

**Figure 1:**
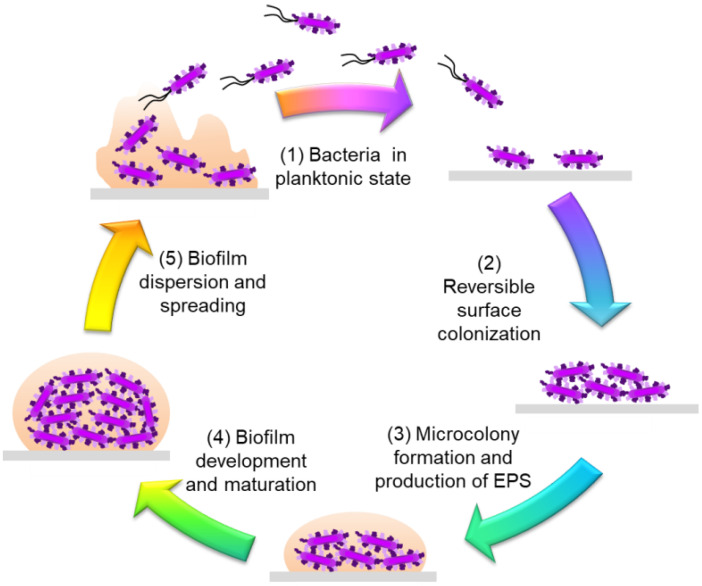
Life cycle of a bacterial biofilm. Planktonic bacteria, free in a medium **(1)** bind reversibly to a surface **(2)**. They secrete adherent proteins as well as EPS resulting in irreversible attachment and form a microcolony **(3)**. The biofilm grows and matures **(4)** until, after an event, the bacteria in the biofilm revert to a planktonic lifestyle **(5)**.

In order to understand the tolerance mechanisms of biofilms, it is important to decipher their life cycles, from their formation to their dissemination. The formation of biofilms takes place in several stages (**Figure 1**).

- Initially, planktonic bacteria, evolving freely in an aqueous environment, attach themselves to a biotic or an abiotic surface. For bacteria to attach, certain structures and proteins must be present on the bacterial surface, such as curli, fimbriae and pili present in E. coli or Salmonella spp. 22 23. Surface coating with an organic conditioning film can alter bacterial initial adhesion and promote biofilm growth 24. Different phenomena are involved in the transport of bacteria to a surface: motility, Brownian motion, gravitation, diffusion, convective transport, etc. 25. This first attachment is reversible and is triggered, when bacteria are between 2 and 50 nm from the surface, by non-specific interactions such as van der Waals, Lewis acid-base and electrostatic interactions 25.
- The attachment becomes irreversible mainly by the formation of adhesins and adhesive proteins, which anchor the bacteria to the surface. By modifying their environment, sensors located in the internal membrane of bacteria can activate a succession of reactions (autophosphorylation, transient protein-protein interactions, enzymatic activity) which leads to cyclic diguanosyl monophosphate (c-di-GMP) synthesis. The intracellular level of c-di-GMP directs the bacteria towards a chronic lifestyle to form biofilms (e.g. if the level is high), or towards an acute lifestyle which corresponds to the bacteria in the planktonic state (e.g. when the level is low) 26. The bacteria change their patterns of gene expression to adopt a biofilm mode of life, for example by triggering EPS production that cements them together 27.
- The next step is biofilm maturation. This is characterized by an increase in size, thanks to the EPS synthesized by the bacteria, and a 3D structure is obtained. The shape depends on the bacterial species and the specific strain involved. The production of large quantities of EPS helps to protect the biofilm and also increases its tolerance to antimicrobial action 28. Gene expression in bacteria present within the complex structure of the mature biofilm is extremely heterogeneous and distinct from that in planktonic bacteria.
- The last step is the dispersion of the biofilm. After the biofilm matures, some detached bacteria may adhere to a new surface and form a new biofilm: this completes the life cycle of the bacterial biofilm. There are several reasons for the return to the planktonic state: a lack of nutrients that causes bacteria to seek a better environment, or an accumulation of toxic residues 28,29. Other reasons for the detachment of bacteria are mechanical disturbances (induced by shearing forces, by abrasion), enzymatic degradation of the polymer matrix (thanks to dispersin B, for example), the production of surfactants (e.g. rhamnolipids in P. aeruginosa), the induction of motility with a new synthesis of flagella, and the release of the EPS 29. The c-di-GMP also plays a role in the dispersion of the biofilm, together with QS which is regulated by the agr gene in S. aureus 30.

Depending on their environment, bacterial biofilms have different growth patterns, different spatial organizations as well as different phenotypic and genetic characteristics 31. Within a single species of bacteria, a great variety of subpopulations can be obtained due to their great genetic plasticity. Division of the biofilm into several populations is affected by environmental conditions: chemical gradients (oxygen, nutrients and electron donors), adaptation to local environmental conditions, gene expression and the genotypic variation that occurs through mutation and selection 32.

### 2.2 Biofilms on artificial surfaces: Medical equipment

Medical equipment, particularly that intended for insertion into the body, provides many opportunities for bacteria to enter the body, to develop biofilms and to proliferate 38. Catheters, implants and prostheses spread biofilms into the blood or other organs, causing inflammation and disease. Preventative treatments of the surface of medical equipment with antimicrobials, or insertion of cement and bone spacers releasing broad-spectrum antibiotics, are required. Despite very strict health protocols, biofilm formation is widespread (**Table 2**).

## 3. Failure of antimicrobial action on biofilm communities

The high tolerance of biofilm bacteria to otherwise lethal conditions and to the immune system is due to different factors: i) the matrix hampers diffusion/reaction of biocides; ii) preferentially expressed genes are involved in stress response or in antibiotic efflux; iii) phenotypic variants with enhanced ability to survive adverse conditions appear 44. However, no current consensus exists regarding the mechanisms of biofilm tolerance, though numerous competing theories are presently under investigation 45. Biofilm-associated persistence is of particular importance in the context of antibiotic multiresistant bacteria, and powerful molecules are actively sought.

### 3.1 Disinfectants, antiseptics and antibiotics

A vast array of compounds, known collectively as antimicrobials, are used to fight bacteria and, more generally, microorganisms. Disinfectants, antiseptics and antibiotics are products with an antimicrobial, therefore antibacterial, antifungal and/or antiviral action. They are intended to inhibit growth (bacteriostatic, fungistatic) and/or kill microorganisms (bactericide, fungicide, virucide). Disinfectants are used on inert materials (medical equipment, floors, etc.). Conversely, antiseptics are used on living tissues, for external use only, and are designed so as not to destroy the tissues to which they are applied. Unlike disinfectants, antibiotics target only bacteria and are given orally, by ingestion or by perfusion. An antibiotic can be both bactericidal and bacteriostatic, depending on its dose. There are several families of antibiotics, with different structures and modes of action. They are grouped according to the mechanism of their action on planktonic bacteria:

- β-lactam family for the inhibition of bacterial wall synthesis.
- aminoglycosides, macrolides, phenicols, cyclins, fusidic acids and oxazolidinones for the inhibition of protein synthesis.
- quinolones and mupirocins for the inhibition of nucleic acid synthesis.
- sulfonamides for folic acid inhibition.
- other antibiotics, such as nitro derivatives and anti-tuberculosis drugs 46.

The minimum inhibitory concentration (MIC) is the standard for determining the sensitivity of planktonic bacteria to an antimicrobial. It represents the lowest concentration of an antimicrobial needed to prevent bacterial growth under specific conditions. Compared to planktonic bacteria cultured in liquid media, bacterial biofilms can survive antibiotic doses up to 1 000 times the MIC 47. The presence of EPS, the spatial organization of the community and the physiological changes produced by biofilm growth greatly enhance the survival of bacteria. Consequently, the MIC is not directly related to the minimal biofilm eradication concentration (MBEC).

**Figure 2:**
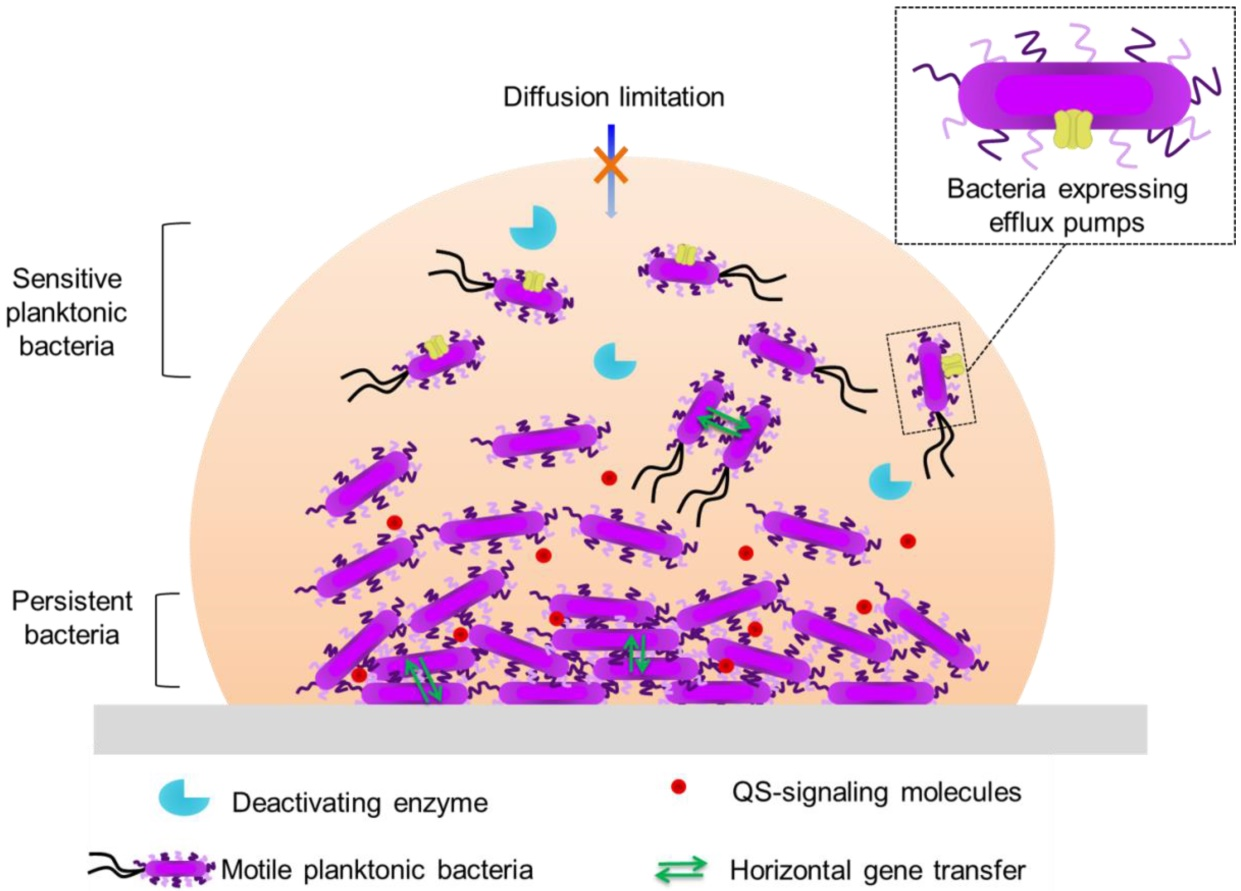
The defense mechanisms of biofilms are numerous and varied: diffusion of antibiotics limited; production of persistent slow-growth bacterial subpopulations; production of enzymes that degrade antibiotics. Bacteria communicate with each other through quorum sensing (QS) and share genetic material by horizontal gene transfer.

Biofilm-associated antibiotic tolerance exhibits certain characteristics that combine with those associated with planktonic bacteria 44: low cell permeability; efflux pumps (rejection of the antibiotic); inhibitory enzymes (β-lactamase) (**Figure 2**).

### 3.2 Antimicrobial diffusion-reaction limitation within biofilms

The matrix acts as a barrier to certain toxic molecules, reducing or preventing their diffusion 44. In *P. aeruginosa* the major component of the matrix is alginate, which is responsible for its anionic character. The EPS matrix of biofilms can prevent antimicrobial interaction with embedded bacteria. The delivery of antibiotics to the bacteria deepest in the biofilm can be compromised, and only the upper layers are exposed to a lethal dose. Bacteria in biofilms formed by *P. aeruginosa* are more tolerant than their planktonic counterparts to several antibiotics (aminoglycosides and β-lactams) partially due to their low membrane permeability and their low proportion of porins on the outer membrane of the cell wall 48.

In addition, in *P. aeruginosa* biofilms, tolerances have developed, with the production of a β-lactamase which lyses the β-lactam ring during its diffusion in the matrix 49. Non-mucous strains form biofilms which have much lower tolerance towards tobramycin, one of the antibiotics most widely used to fight *P. aeruginosa* 50. Nevertheless, β-lactam antibiotics can penetrate the biofilm, and it appears that biofilm tolerance is not really related to the matrix interference 51.

The matrix of *S. epidermidis* interferes with the sensitivity to a large number of antimicrobials 52. Not all activities are equally reduced; glycopeptides such as vancomycin and teicoplanin are significantly affected, and one hypothesis is that this is due to their high molecular weights, 1 450 and 1 600-1 900 g/mol, respectively. Agents such as rifampicin, clindamycin and macrolides, with molecular weights from 360 to 830 g/mol, are either unchanged or little affected. However, this hypothesis is challenged, because daptomycin (MW = 1 619 g/mol) is not significantly perturbed by the matrix. Another hypothesis is that the matrix itself interferes with the local defenses of the host and creates conditions of local immunosuppression.

Several advanced fluorescence microscopic tools show that the phenomenon of diffusion in the matrix is not sufficient to explain the resistance of biofilms to antibiotics. Confocal time-lapse imaging, FLIM, FRAP, and FCS methods demonstrate that vancomycin penetrates the *S. aureus* biofilm matrix within 2 to 3 minutes (depending on the strain) 53. This slow diffusion is explained by the interaction between the cationic vancomycin and the anionic biofilm components (cell surface of the bacteria, pocket of eDNA…). Only between 50 and 60% of the vancomycin is able to diffuse freely in the biofilm. This retention of antibiotics impacts bioavailability, making them less effective on biofilms.

Daptomycin is an antibiotic that works by creating large pores through the membranes of cells, breaking them down, causing potassium ions to leak, thereby killing bacteria. The failure of daptomycin to treat *S. aureus* biofilms was considered, in early studies, to be due to its poor diffusion within the biofilm. However, it has since been shown to diffuse into the biofilm and interact with the bacteria therein, but oligomerization allowing pore formation does not occur 54. The explanation lies in the composition of the cell membrane, and more precisely in the nature of the fatty acids they contain. In fact, the bacteria in *S. aureus* biofilms produce more saturated fatty acids than planktonic bacteria, thus increasing membrane rigidity, limiting exchanges and therefore constituting a defense mechanism against foreign elements.

The action of tobramycin against *Acinetobacter baumannii* and *S. aureus* biofilms is limited by adsorption at the binding sites of the matrix surface 55. The diffusion of five anti-pseudomonas antibiotics (ceftazidime, cefsulodin, piperacillin, gentamicin and tobramycin) through alginate gels was studied 56. β-Lactams diffuse through the matrix faster than aminoglycosides which are hydrophilic and positively charged; these initially bind to alginates, but diffusion increases after a latency period of 80 to 100 minutes. This has inspired the use of cationic compounds such as quaternary ammonium compounds (QACs) to destabilize the biofilm; these also have bactericidal properties. However, QACs are generally not very selective and are therefore very toxic. Short alkyl chains cannot cross the surface of the biofilm. QACs with C8 and C10 alkyl chains enter water channel systems easily; this is facilitated by convective flow and there is no loss of their bactericidal activities. However, a further increase in the hydrophobicity leads to a gradual loss of activity 57. QACs diffuse poorly in *P. aeruginosa* biofilms and therefore are not very effective 58. Their effect is correlated with the composition of the biofilm matrix, as well as its thickness.

### 3.3 Biofilm-specific Metabolism and Physiology

Within the biofilm, several subpopulations are formed with an increased ability to survive difficult conditions. Bacteria, called persisters, are stress-tolerant microorganisms that do not die or grow in the presence of multiple antibacterials 59. These are found in several species of bacteria such as *E. coli*, *P. aeruginosa*, *S. aureus*, and *Gardnerella vaginalis.* They are largely responsible for the persistence of chronic infections related to biofilms. These bacteria are said to be in a state of dormancy, where their metabolism is inactive and there are no genetic changes. The availability of nutrients is a key factor in determining the metabolism of the biofilm 60. A very slow metabolism can limit the activity of many antimicrobial compounds. When an antibacterial is applied, the persisters are not sensitive and, when the level of biocide decreases, they can repopulate the biofilm. This tolerance to antibiotics is not genetic, but stems from the fact that their slow-growth state protects them. The level of persistence depends on the antibiotic used as well as the level of biofilm maturation.

The local oxygen level greatly influences the tolerance of *P. aeruginosa* to antibiotics 16. This is because tobramycin and ciprofloxacin are able to penetrate the biofilm matrix, but can only kill bacteria at the air-biofilm interface. The oxygen-rich zone coincides with the region of active metabolism in the biofilm. In the absence of oxygen, these antibiotics are unable to kill bacteria embedded deep in the biofilm. This shows that limited antibiotic diffusion is not the primary protective mechanism for these biofilms, because in this case the tolerance to antibiotics is related to the oxygen levels. Results for artificial *E. coli* biofilms against two antibiotics, latamoxef and tobramycin, are similar 16.

### 3.4 Genetic plasticity

The expression of certain genes allows biofilm bacteria to survive in response to stress, to the introduction of antibiotics or to the failure of cell-to-cell communication (**Figure 2**). A number of genes, as well as regulatory processes, are activated during biofilm formation, particularly during the adhesion, growth and maturation stages. For *P. aeruginosa,* the biosynthesis of alginate is encoded by the *algC* gene, the expression of which is triggered by QS 61. For *S. aureus*, the *agr* gene, which drives biofilm formation, is also activated by QS 33.

In response to the introduction of an antibiotic, multi-drug efflux pumps can be overexpressed at the surface of the exposed bacteria 62. There are several families of pumps, the best known and studied being the resistance-nodulation-division (RND) family. This efflux pump has three components: a cytoplasmic inner-membrane pump (AcrB protein), a periplasmic adapter protein (AcrA) and an outer-membrane protein channel (TolC). The pump crosses the wall of Gram-negative bacteria, and allows the expulsion of a broad range of antibiotics. In *P. aeruginosa* biofilms, when an antibiotic is introduced, the efflux pumps increase their activity 63. When the concentration of the antibiotic is below the inhibitory threshold, alginate genes are expressed 64. This reaction in response to antibiotic stress is present in biofilms formed by *E. coli*, where there are genes associated with multidrug efflux pumps. In fact, the production of biofilms is significantly slowed down when certain genes are removed by mutation 65. When these pumps are deactivated (using an inhibitor molecule), biofilm growth is reduced and the tolerance towards antibiotics is blocked 66. This demonstrates that efflux pumps are a mechanism of bacterial biofilm resistance (**Figure 2**).

Generally, several species of microorganisms are present within biofilms. This allows the sharing of genetic material by horizontal gene transfer 67. Biofilms provide the ideal conditions for such transfer, such as high local cell density, accumulation of genetic elements from different bacteria and elevated mutation frequencies. This transfer of information is conducive to the acquisition of new traits and to increased resistance. Gene transfers have been identified and described in a variety of biofilms 68. The density and age of the biofilm have an influence on the efficiency of gene transfer.

## 4. Alternative therapies for biofilm eradication

Due to the failure of most antibiotics to treat biofilms, alternative therapies have been sought in order to eradicate or to inhibit them. Several points of intervention are possible, from the formation of the biofilm until its maturation. Many natural products hinder QS, have anti-adhesin activity, inhibit film growth or have non-specific antimicrobial properties. Other therapies use bacteriophages, viruses which specifically target certain bacteria, or enzymes which degrade the extracellular biofilm matrix.

### 4.1 Natural products

Some natural antibiofilm agents are able to inhibit or drastically reduce QS signaling. Among them, garlic extracts render *P. aeruginosa* sensitive to tobramycin as well as to phagocytosis by polymorphonuclear leukocytes, by reducing signal production 69. By studying the critical interactions within the binding site and structural motifs in the isolated molecular components of garlic, it has been possible to design compounds that inhibit QS; examples include: citrus limonoids, isolimonic acid and hordenine, an extract from barley kernels 70. *Chamaemelum nobile* is a plant widely used for its anti-inflammatory, deodorant, bacteriostatic, antimicrobial, carminative, sedative, antiseptic, anticatarrhal and spasmolytic properties. It inhibits QS in *P. aeruginosa* resulting in inhibition of biofilm formation and growth 71.

The ethyl acetate-soluble fraction of *Cocculus trilobus* has anti-adhesin effects at the adhesion stage of biofilm formation from Gram-positive bacteria 72. Polyphenols from cranberries have effects on the formation, growth and adhesion to surfaces of *Porphyromonas gingivalis* biofilms 73. They also inhibit the adhesion of *E. coli*, inhibit the sialic acid-specific adhesion of *Helicobacter pylori* 74, and prevent biofilm formation of *Streptococcus mutans* 75 and *Streptococcus sobrinus* 76. *Herba patriniae*, a traditional Chinese medicinal herb, inhibits the expression of six key genes associated with *P. aeruginosa* biofilm formation and EPS production 77. Other molecules such as ginkgolic acids, extracted from the *Ginkgo biloba* tree, have neuroprotective, antimicrobial, and antitumor properties 78, and are active against biofilm formation in *E. coli* and on three strains of *S. aureus* 79.

Manuka honey, native to New Zealand, and Sidr honey, native to Yemen, have bactericidal efficacy, as well as antibiofilm activity 80. This use of honeys is particularly interesting in that they are natural and non-toxic products. These honeys can destroy *in vitro* planktonic bacterial cultures and biofilms of 11 strains of *S. aureus* sensitive to methicillin (MSSA), 11 strains of *S. aureus* resistant to methicillin (MRSA) and 11 strains of *P. aeruginosa* (**Table 3**). This is better than most commonly used antibiotics.

Indeed, of 9 antibiotics (rifampin, cefazolin, oxacillin, vancomycin, azithromycin, fusidic acid, gentamicin and linezolid) of different classes, and used routinely against *S. aureus*, only rifampin eradicates MSSA or MRSA biofilms 81. Rifampin is bactericidal for 18% of MSSA and 42% of MRSA, which is significantly poorer than for the two honeys. The active ingredient of Manuka honey is methylglyoxal, also known as pyruvaldehyde or 2-oxopropanal 82. It is present at a concentration 100 times greater than in other honeys, where the bactericidal activity is due to *in situ*-generated hydrogen peroxide 83, the amount varying with the species. Being a powerful oxidant and reducing agent, H_2_O_2_ denatures the proteins of microorganisms.

### 4.2 Bacteriophages

Alongside natural products, other alternative therapies which mimic strategies observed in nature have been designed in order to destroy bacterial biofilms. Phage therapy is based on the use of bacteriophages, viruses that specifically infect bacteria. These viruses do not infect eukaryotic cells, thus reducing the risk of opportunistic infections. Their average size ranges from 25 to 200 nm, and they consist of a protein capsid protecting their genome (containing nucleic acid), most often a tail of variable length (through which the genetic material is injected) and of tail fibers ensuring recognition of the host (pili can be receptors) 84. Bacteriophages are able to alter polysaccharides in biofilms using enzymes, called depolymerases, located at the ends of the tail fibers 85, increasing their diffusion into the matrix of the biofilm and hence their efficacy. Moreover, bacteriophages can diffuse freely through the water channels of the biofilm 86. Bacteriophages are very specific to a species subgroup population and do not have such a broad spectrum of action as antibiotics.

In the same way as for planktonic cultures, phage-resistant subpopulations can emerge within the biofilm community 87. Phage-resistant mutants of *P. aeruginosa* biofilm appear after treatment with anti-pseudomonas bacteriophages, due to the mutation of genes encoding the phage receptors 88. QS is related to resistance against bacteriophages in biofilms: *E. coli* reduces the number of receptors on the cell surface in response to AHL detection signals, resulting in a two-fold reduction in the rate of phage adsorption 89.

During the 20th century, the Soviet Union (USSR) invested in bacteriophages to treat bacterial infections. Not being able to afford antibiotics, produced mainly by western countries, the USSR focused on other means of treating their populations. For this reason, in Georgia, formerly part of the USSR, the Eliava Institute now has one of the largest libraries of bacteriophages, having started its research in 1923, just a few years after their discovery 90.

### 4.3 Biofilm-dispersing enzymes

Due to its porous structure, as well as its exposure to the surrounding environment, the biofilm matrix is an attractive target for antibiofilm therapy. Certain enzymes are able to degrade the polymers of the biofilm matrix. This prevents their formation, loosens the matrix formed on a surface and increases the sensitivity of the bacteria in the biofilms to antibacterials.

One of these enzymes is Deoxyribonuclease I (DNase I). Its antibiofilm action was tested on Gram-positive bacteria (Enterococcus faecalis, S. aureus, Staphylococcus epidermidis, Staphylococcus haemolyticus, Streptococcus intermedius, Streptococcus mutans, Streptococcus pneumoniae and Streptococcus pyogenes), and Gram-negative bacteria (Acinetobacter baumannii, Aggregatibacter actinomycetemcomitans, Bdellovibrio bacteriovorus, Campylobacter jejuni, Comamonas denitrificans, E. coli, Haemophilus influenzae, Klebsiella pneumoniae and P. aeruginosa) 91. It inhibits the formation of biofilms of 7 species out of the 17 tested, can partially or totally detach biofilms from 15/17; and makes 7 out of 17 species sensitive to antibacterials. This enzyme also induces biofilm dispersion in Bordetella bronchiseptica, Bordetella pertussis and Gardnerella vaginalis.

Pulmozyme® is a drug marketed in France in the form of a solution by the ROCHE laboratory 92. Based on DNase I it is used to treat *P. aeruginosa* infections in patients with cystic fibrosis. This drug exhibits biofilm detachment activity against established *P. aeruginosa* and *S. pneumoniae* biofilms *in vitro*.

Dispersin B is produced naturally by *A. actinomycetemcomitans*. This enzyme hydrolyzes an extracellular polysaccharide, poly-(β-1,6)-*N*-acetylglucosamine (PNAG), produced by several bacteria including *S. epidermidis*, *S. aureus* and numerous members of the Gram-negative *Proteobacteria* family. Degradation of the biofilm matrix triggers its dispersion and resensitizes residual bacteria to the action of an antibiotic 93.

Another strategy is to use enzymes which are part of EPS synthetic pathways, such as alginate lyase and PelA. Alginate lyase hydrolyzes alginate, one of the components of the extracellular matrix of biofilms, thus disrupting its structure. It reduces the viscosity of sputum in cystic fibrosis, improves phagocytosis and increases the effectiveness of antibiotics. PelA is a protein necessary for the synthesis of the polysaccharide Pel, which is a component of the biofilm matrix 94. Pel promotes cell-cell interaction in the biofilm structure, and plays a role in cell adhesion to a surface. It also protects bacteria against certain aminoglycoside antibiotics. PelA can hydrolyze Pel, control its length and rectify errors in the case of malformation. It can inhibit the formation of biofilms of *P. aeruginosa* as well as *Pseudomonas spp*., and in the latter, can destroy an already formed biofilm.

## 5. Contribution of nanotechnology against biofilms

**Figure 3:**
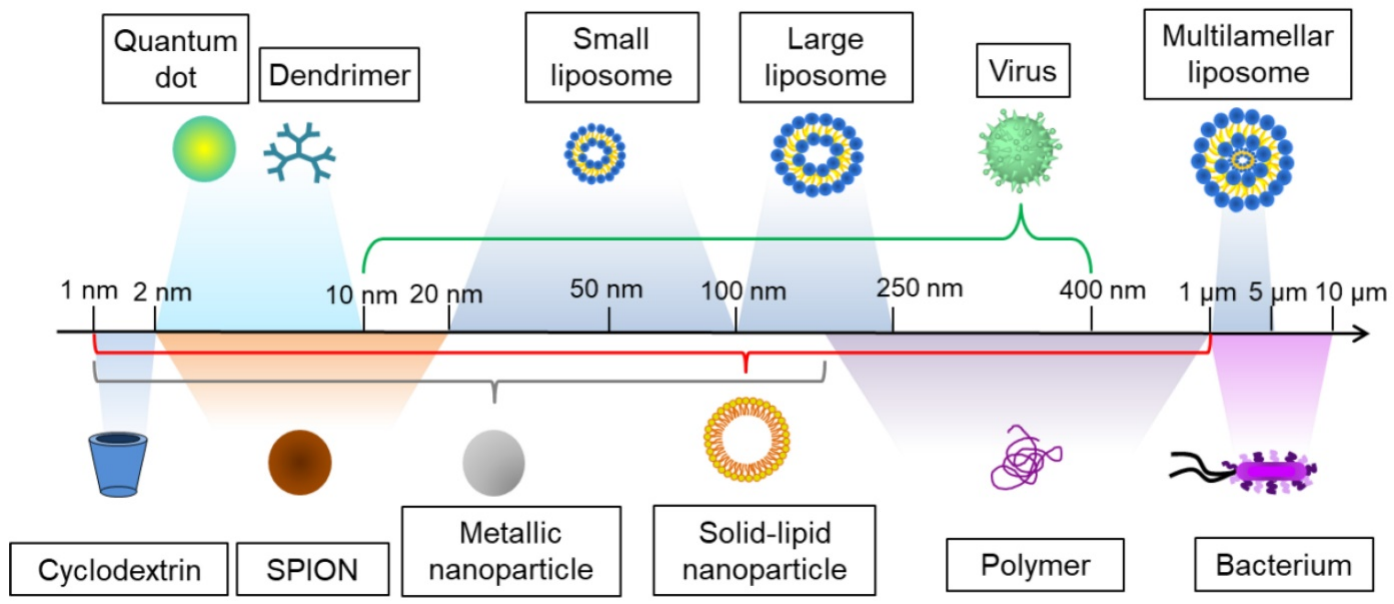
The relative sizes of several nanomaterials are shown here ranging from 1 nm to >1 µm. Superparamagnetic iron oxide nanoparticles (SPION).

In parallel with the classic methods for treating bacterial biofilms, new technologies have emerged. Nanotechnology can be defined as the design and study of materials at the nanometer scale, typically between 1 and 1 000 nm (**Figure 3**). The nanoscale character results in unique properties, both physicochemical and biological. This is due to their large surface-to-volume ratios, which confer properties different from those of the bulk. The applications of nanotechnologies are very diverse and numerous, in health 95, energy 96, defense 97, environment 98, information storage 99, etc.

Nano-formulations have several advantages in the field of drug delivery in that they allow:

- administration of drugs that are poorly soluble in water;
- protection of the drug from enzymatic reactions;
- targeting of drugs to the specific organ to be treated, thereby reducing potential toxicity;
- crossing of several membranes impermeable to traditional medicines;
- intracellular and transcellular delivery of large macromolecules.

Since the pore diameter of biofilms averages about 50 nm (this value depends on the density of the biofilm) 100, nanomaterials, with sizes below this value, can diffuse through the biofilm matrix and easily reach bacteria in the inner regions of the biofilm. The biokinetics of encapsulated drugs is different from that of free drugs, focusing antibiotic action on the biofilm and minimizing exposure to human cells. Nanomedicines can increase efficiency, specificity, and biodistribution, as well as reducing dosages and thus the toxicity of the corresponding drug formulations.

The surface functionalization of nanomaterials also plays a role in diffusion within the biofilm. Positively charged nanomaterials better penetrate biofilms having a negatively charged matrix, and hydrophobic particles have a better distribution within biofilms than hydrophilic ones 101. In addition, the physical properties of nanomaterials can be exploited to fight biofilm. The intrinsic bacterial toxicity of some inorganic nanomaterials, or the capacity of some nanomaterials to locally induce heat, can be harnessed to cause bacterial death. In the constant search to improve treatments while reducing the doses administered, the idea of ​​a multifunctional nanomaterial combining therapy and diagnosis (theranostics) has emerged.

Diagnostic techniques using nanomaterials as biomarkers are widely used in medical imaging. Depending on the technique used, different information can be obtained. By means of nanomaterials it is possible to perform structural imaging, to obtain a 2D or 3D image of an organ, with information about its size, volume and the location of any visible abnormalities (tumors, for example). Different methods are based on:

- magnetic fields (magnetic resonance imaging (MRI), magnetoencephalography (MEG), etc.)
- X-rays (radiography, X-ray-computed tomography, two-photon X-ray absorption (DEXA))
- ultrasound
- light rays (diffuse optical tomography, near-infrared spectroscopic imaging)
- radioactivity (positron emission tomography (PET), single-photon-emission-computed tomography (SPECT))

Functional imaging gives quantitative information about biological parameters, metabolism in particular. Usually only one type of imaging is used. However, it is possible to combine them in bimodal imaging, which allows microscopic and macroscopic information to be obtained from two imaging techniques with a single probe. Among the most common are the combinations of:

- MRI and PET scans
- MRI with optical imaging (fluorescence, bioluminescence)
- MRI and photoacoustics
- PET and computed tomography (CT)

**Figure 4:**
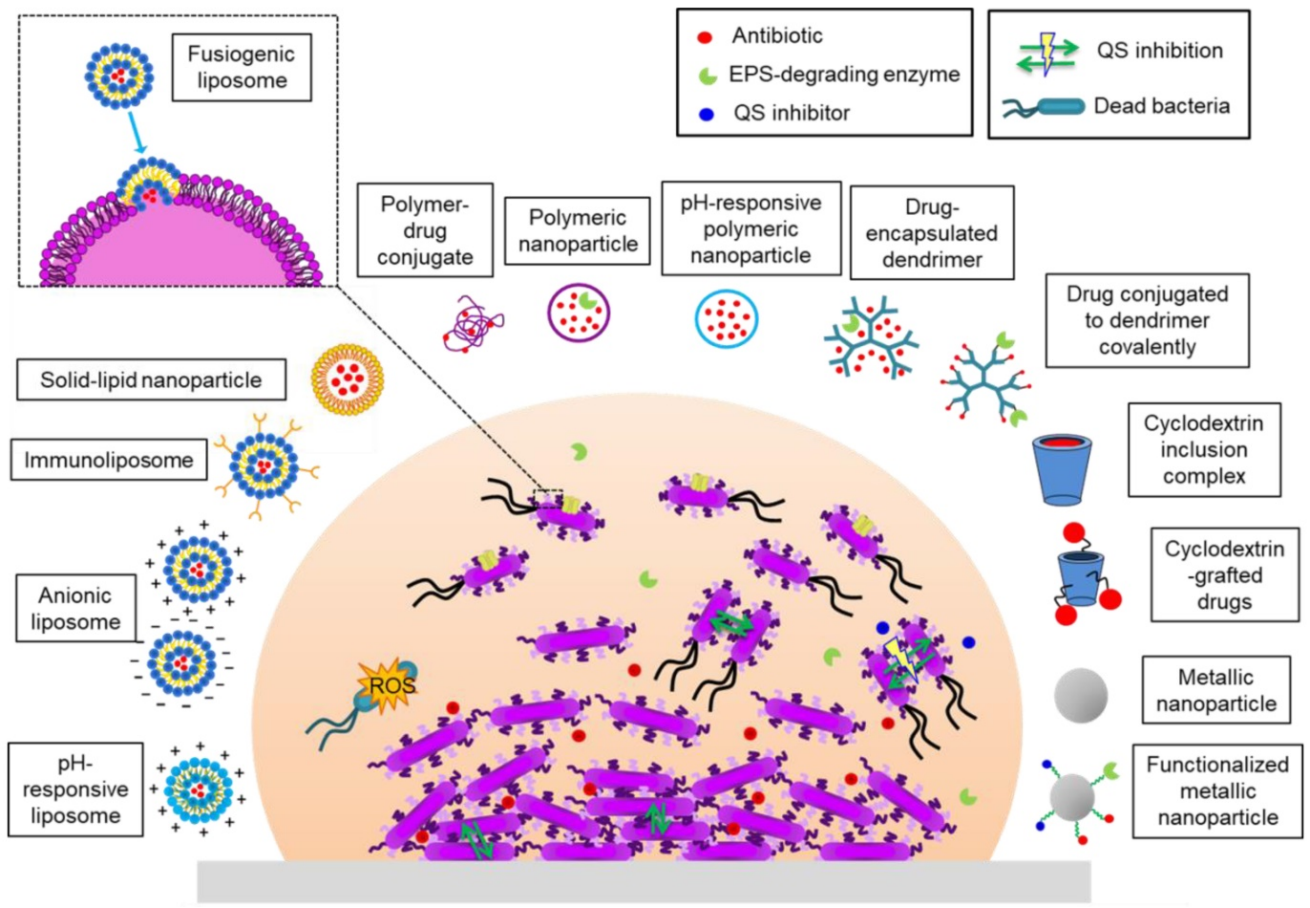
Nanomaterials with activity against biofilms.

**Figure 5:**
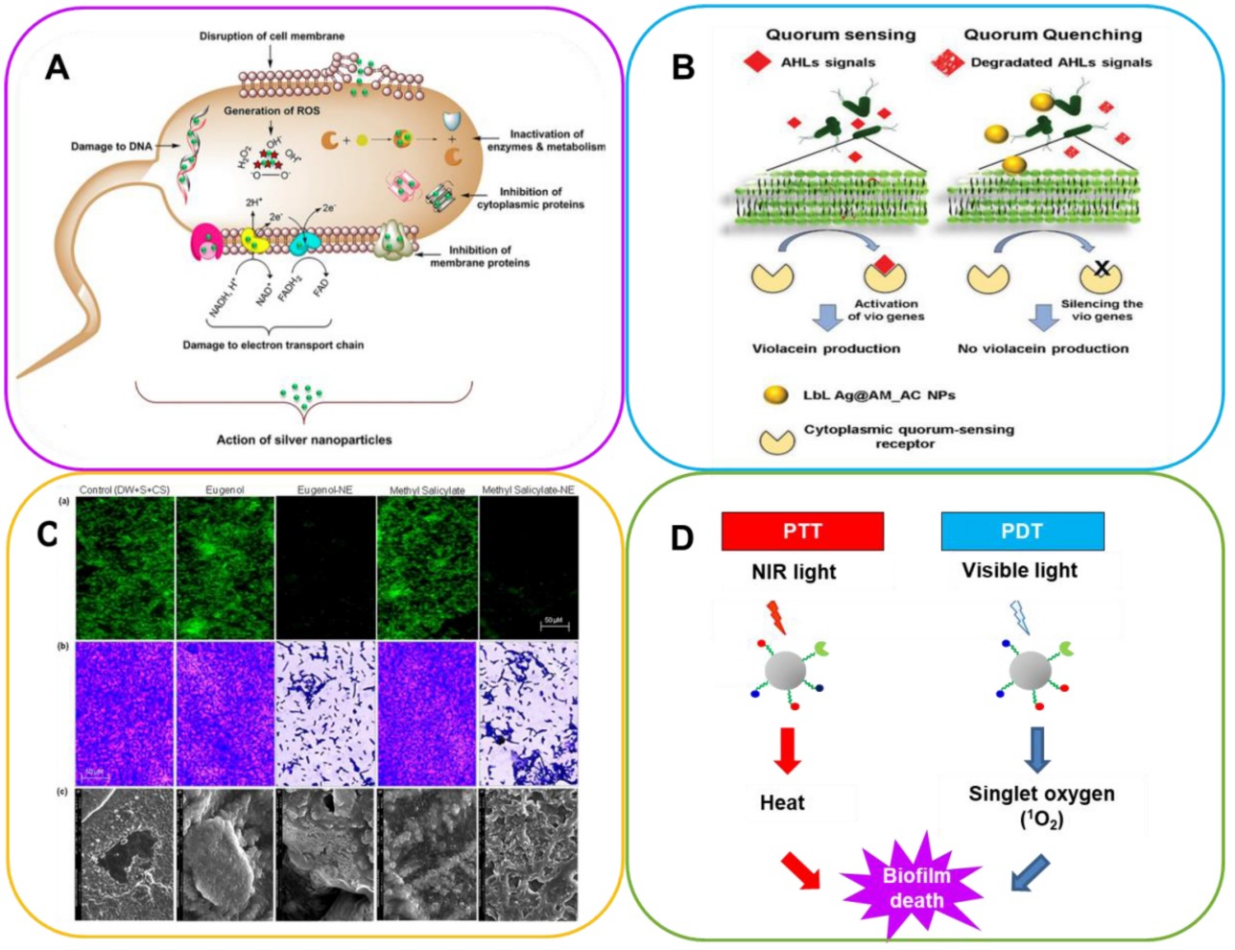
**A.** Mode of action of Ag-NPs on cell apoptosis. Adapted with permission from 102, copyright 2020 Scientific Reports. **B.** AHL signals bind to the Quorum Sensing receptor and switch on vio genes, activating violacein production (**B.** left). The presence of Quorum Quenching-active NPs breaks down AHL signals, silencing vio genes, thus inhibiting violacein production (**B.** right). Adapted with permission from 103, copyright 2020 Advanced Functional Materials. **C.** Inhibition of *E. coli* biofilm formation after exposure to nanoemulsion-loaded hydrogel coatings containing eugenol-NE and methyl salicylate-NE for 7 days on solid surfaces, namely (**a**) glass, (**b**) plastic and (**c**) meat, measured by fluorescence microscopy, phase contrast microscopy and SEM, respectively. Adapted with permission from 104, copyright 2019 Scientific Reports. **D.** Differences between PTT and PDT using functionalized nanoparticles.

Several nanomaterials have been devised and tested in order to eradicate bacterial biofilms (**Figure 4**). They can be divided into two groups: (i) organic nanoparticles including liposomes, polymeric nanoparticles (NPs), dendrimers, cyclodextrins and solid-lipid NPs; (ii) inorganic NPs which include metallic NPs (gold, silver, silica, copper, etc.), metal oxides (iron oxide, aluminum oxide, etc.), quantum dots, fullerene and organic-inorganic hybrids. In some systems bactericidal activity is due to the nature of the nanomaterial or the nanocarrier. Others allow a drug to be encapsulated, protecting it from enzymatic deactivation and environmental conditions that can compromise its action (lack of oxygen, pH) and the defense mechanisms of the bacteria (**Figure 5**) 102-104.

### 5.1 Drug delivery

Different nanomaterials have been applied for the delivery of antibiotics to biofilms, aiming for a more effective delivery of the drug in the biofilm, or a targeted delivery restricted to the bacteria, reducing side-effects in human tissue. We will review the most important nanomaterials used so far for antibiotic delivery.

#### 5.1.1 Liposomes

A liposome is an artificial spherical vesicle, made up of at least one lipid bilayer, trapping a watery compartment inside. Such a structure facilitates the encapsulation of a wide variety of drugs/active molecules: hydrophilic in the aqueous internal compartment, lipophilic within the lipid bilayer, and amphiphilic between these two regions. Its biocompatibility and its capacity to reduce drug toxicity for *in vivo* applications make it a great platform in the medical field for the vectorization of molecules of therapeutic interest 105. The encapsulation of vancomycin in fusogenic liposomes (which can fuse with the bacterial membrane) increases its bactericidal activity against biofilms of *S. aureus* 106. Cationic liposomal capsules have been developed in order to penetrate the anionic matrix of *S. aureus* biofilms 107. These drug-containing liposomes are more effective than the drug alone, being able to better slow down and inhibit bacterial biofilm growth. The use of liposomes as drug-carriers allows the internalization of drugs by endocytosis. Liposomes being biocompatible, they facilitate the penetration of mature biofilms and enhance the interaction with the membrane of the bacteria. Modifying the liposome surface by grafting a molecule recognized by the biofilm makes a specific interaction possible. By grafting an antibody to the liposome surface, a retention immunoliposome is created, which enables release of the encapsulated drug close to the surface of the biofilm, thus increasing its efficiency 108. A disadvantage, especially with liposomes smaller than 100 nm, is their poor physicochemical stability (spontaneous fusion), leading to a loss of payload and an increase in the size of the vesicles (therefore preventing the crossing of certain barriers). This thereby eliminates the potential therapeutic benefits of nanoscale delivery.

#### 5.1.2 Solid-lipid nanoparticles

Solid-lipid NPs (SLN) are spherical colloidal nanomaterials, with diameters typically between 10 and 1 000 nm 109, composed of a lipid core stabilized by surfactants. SLNs are an excellent antimicrobial drug delivery platform because their small size and lipid core provide good drug protection to ensure maximum bioavailability, therapeutic targeting, better biodegradation and nontoxicity. SLNs combine the advantages of lipid emulsion and polymeric NPs while overcoming temporal stability, storage, cost and *in vivo* issues that hamper drug delivery 110. They are capable of encapsulating lipophilic and hydrophilic drugs, and the carrier has no intrinsic biotoxicity, being composed of lipids similar to physiological ones. It is also possible to freeze-dry and reconstitute SNLs, and they have a high drug payload 111. Their large-scale production reveals no problems; they can be synthesized without organic solvents and they are sterilizable (in an autoclave) 109, making them very interesting for formulations in many treatments.

The antibacterial and antibiofilm activity of a complex composed of SLN encapsulating cefuroxime axetil (CA), a 2^nd^ generation cephalosporin, was tested against *S. aureus* 112. Encapsulation allows prolonged release: it takes 12 hours to achieve 96% release of CA encapsulated in SLN as against 99.7% in 2 hours if it is free. This complex forms an inhibition zone to biofilm formation, larger than CA alone (13 mm *vs*. 9 mm) and has a lower MIC against *S. aureus*.

Several QS-inhibitor (QSI) nanomaterials have been designed to eradicate or inhibit the formation of biofilms 113. They have many advantages over conventional compounds: nanomaterials are able to penetrate the biofilm matrix, have better solubility, can deliver drugs efficiently and specifically, as well as maintaining the activity of the nano-inhibitor (no agglomeration or aggregation). A QSI encapsulated in an ultra-small (diameter < 100 nm) solid-lipid NP (us-SLN) shows up to seven times more activity than the free QSI alone in the eradication of *P. aeruginosa* biofilms 113.

#### 5.1.3 Polymeric nanoparticles

Polymeric NPs (PNPs) constitute another type of nanotechnology developed to fight bacterial biofilms. They can be defined as solid colloidal particles with sizes less than 1000 nm. PNPs increase the solubility index of a large number of molecules, hydrophilic and hydrophobic, and can be made out of biodegradable polymers. PNPs have been applied for antibiotic delivery, especially for hydrophobic drugs. An antibiotic, levofloxacin, encapsulated in a poly(lactic-co-glycolic acid) (PLGA) nanocapsule, shows antibiofilm activity against *E. coli* bacteria. It destroys the biofilm and inhibits the growth of surviving bacteria 114.

#### 5.1.4 Dendrimers

Dendrimers are 3D, branch-shaped structures with repeating molecular patterns. They are of interest for drug delivery due to their ability to encapsulate both hydrophilic and hydrophobic molecules in the free spaces between their branches 115. It is also possible to graft molecules of therapeutic interest to these polymers, through functional groups at the surface of the dendritic structure. Some peptide-grafted dendrimers form a thermally reversible collagen-type triple helix. This allows them to act as drug transporters, with delivery capabilities under heating. Dendrimers are also widely used as vehicles for transporting DNA and anti-cancer drugs 116. Low-molecular-weight peptide dendrimers express antimicrobial properties, without any antibiotics, against *S. aureus* and *E. coli* 117.

#### 5.1.5 Cyclodextrins

Cyclodextrins (CD) are cyclic oligosaccharide compounds, composed of glucose units linked together by α-1,4-glycosidic bonds. There are three main types of CD, differentiated by the number of glucose units, namely α-CD (6 glucose units), β-CD (7 units) and γ-CD (8 units). They are used in various fields, in food 118, in the environment 119, in pharmaceuticals 120 as well as in drug delivery. The hydrophobic interior of CDs enables complexation with hydrophobic drugs inside the macrocycle, creating supramolecular structures. This makes them very good solubilizers and stabilizers, and allows the transport of the drugs to the target 121. CDs increase the solubility of a drug by encapsulating it, and protect it from the degradation reactions that can occur with oral drug administration. There are more than 30 pharmaceuticals using CD-drug complexes in the world 122. Drugs encapsulated in CDs, covalently attached to a surface, have antibiofilm activity against *C. albicans*. Encapsulation of anidulafungin or thymol in several CDs, attached to a gold surface, shows inhibition of *C. albicans* adhesion, to the extent of 64% and 75%, respectively 123. Likewise, encapsulation of miconazole in CD, attached to polyethylene and polypropylene surfaces, reduces *C. albicans* biofilm formation by 87 to 96% 124.

Another example is the encapsulation of rifabutin (RFB). This drug has only a 20% biodistribution 125, the rest being eliminated through the gastro-intestinal tract. The β-CD-RFB complex eradicates biofilms of *S. aureus*, *P. aeruginosa*, *E. faecalis* and *P. vulgaris* 126. In the absence of β-CD, with RFB alone, inhibition of the biofilm occurs but the remaining bacteria aggregate into large agglomerates. With the β-CD-RFB inclusion complex, inhibition is more efficient.

#### 5.1.6 Hydrogels

Hydrogels are increasingly finding applications in the field of biofilms and wound dressing due to their excellent biochemical and mechanical properties. Several categories of hydrogels have been used to eliminate mature biofilms and progress has been made in related drug-delivery nanosystems. Some of them are based on polyethylene glycol dimethacrylate tethered with a dangling polyethylenimine 127, bioactive essential oil components 128, self-adapting chitosan 129, etc. Drugs, bacteriophages and other active molecules can be encapsulated inside hydrogels, and the rate of release controlled by the gel formulation. Degradation of hydrogel in the presence of infected cells leads to release of active molecules into the infection site, killing bacteria and leading to biofilm eradication, as in the case of *P. aeruginosa, S. aureus, Acinetobacter baumannii*, etc. Hydrogels are promising biomaterials to fight against multidrug-resistant bacterial infections. They have potential in the treatment for biofilm-associated wound infections and enhance healing 130.

### 5.2 Nanomaterials with intrinsic antibacterial properties and stimuli-responsive nanomaterials

#### 5.2.1 Inorganic nanoparticles

Inorganic NPs (INPs) are particles with one or more dimensions (length, width, and thickness) in the range of 1-100 nm, composed of either pure metals (for example, gold, silver and iron), metals oxides or metals salts. The main characteristics of INPs are a large surface area-to-volume ratio compared to their bulk equivalents, large surface energies, plasmon excitation, quantum confinement, a large number of poorly coordinated sites such as corners and edges, specific chemical properties and the ability to store excess electrons. INPs are widely used in imaging as contrast agents, in diagnostics as bio-sensors using their optical properties, as well as therapeutic agents which exploit their thermal, magnetic, and drug delivery properties.

Metallic NPs (MNPs) are characterized by a phenomenon called surface plasmon resonance (SPR), which is the collective oscillation of free (valence) electrons when they are excited by light. This effect gives them excellent optical properties and induces a strong enhancement of the electric field at their surface leading to several possible applications: sensors, *in vivo* bio-imaging, therapeutics, etc. The most interesting metals using SPR are gold, silver and copper.

MNPs are widely used in the medical field, due to their antimicrobial effects on a large number of bacteria, viruses and fungi. Silver NPs (Ag-NPs) are widely used as a bactericide, while gold NPs (Au-NPs) have many catalytic and therapeutic applications. The main mechanisms of their toxicity involve the production of reactive oxygen species (ROS) and impairment of membrane function, for example, with dysfunctional proteins 131. ROS include superoxide radicals, hydroxyl radicals, hydrogen peroxide and singlet oxygen, which may cause chemical damage to proteins and DNA in bacteria. An imbalance between the production of ROS and the antioxidant capacity of the cell is referred to as oxidative stress.

Gold NPs, functionalized with the enzyme proteinase-K (PK), are active against *P. fluorescens* biofilms 132. PK disrupts the matrix, disperses it and destroys bacteria released from the biofilm. The combination of Au-NPs and PK shows a synergistic effect. Au-NPs functionalized with a cationic ligand also show antibiofilm activity in the disruption of the biofilm matrix of *S. aureus* and *P. aeruginosa* 133. Au-NPs inhibit the formation of *C. albicans* biofilms by more than 80% 134, but this is neither due to the generation of ROS nor to the breakdown of EPS from the matrix. Au-NPs bind to the surface of bacteria by strong electrostatic interactions which interfere with those between the pathogens, which are bound by adhesins, thus disrupting and preventing the growth of the biofilm. The growth of *P. aeruginosa* and *S. aureus* biofilms is reduced by the action of Au-NPs 135. At low Au-NP concentration, the growth of *S. aureus* is reduced by 13%, but that of *P. aeruginosa* increases by 3%. At higher concentrations, Au-NPs reduce the concentration of *S. aureus* by 67-70% and that of *P. aeruginosa* by 25-78%. Au-NPs synthesized from rhizome extracts of *Rhodiola rosea* inhibit the formation of *P. aeruginosa* and *E. coli* biofilms. This inhibition is particularly interesting, since Au-NPs do not have a direct bactericidal effect on *P. aeruginosa* and *E. coli* bacteria.

Ag-NPs are known for their broad-spectrum antimicrobial properties, and are also effective against multiresistant bacteria 136, HIV 137 as well as SARS-CoV and SARS-CoV-2 138. Their toxicity in humans limits their therapeutic use, and they also tend to aggregate. The surface functionalization of Ag-NPs improves their antimicrobial effectiveness by preventing aggregation, improving their bactericidal effect within biofilms and decreasing their cytotoxicity 139. Smaller Ag-NPs show better antibacterial activity 140. The shape of Ag-NPs has also an impact on toxicity: triangular NPs are more bactericidal than spherical or rod-shaped ones 141. In aqueous environments, Ag-NPs release Ag^+^ cations: 12% of the silver atoms are in ionic form; thus, the antimicrobial effect cannot be attributed unambiguously to Ag-NPs or to dissolved Ag^+^ ions 142. The antibacterial and antibiofilm mechanisms of action are not yet fully understood. Ag-NPs induce oxidative damage under the effect of stress resulting from the production of ROS 143. Another explanation of the antibacterial action is the ability of Ag-NPS to influence the permeability of bacterial membranes 144 as well as to interact with their proteins, DNA and enzymes, thus leading to genetic mutations, structural alterations and cell division, triggering bacterial death 145.

Ag-NPs in combination with antibiotics have better antibiofilm activity than either alone. Different antibiotics (ampicillin and vancomycin) coupled to Ag-NPs have antibiofilm activity on Gram-negative bacteria (*P. aeruginosa* and *Shigella flexneri*) and on Gram-positive bacteria (*S. aureus* and *Streptococcus pneumoniae*) 146. Synergy is also found between Ag-NPs and polymyxin B, an antibiotic, against *P. aeruginosa* biofilms 147, of which 83% of the samples tested come from multidrug-resistant strains. This combination inhibits the formation of biofilms three times more effectively than the components alone. Of six conventional antibiotics (ampicillin, clindamycin, gentamicin, cephalexin, vancomycin, erythromycin) tested in combination with Ag-NPs against biofilms formed by MRSA 148, the greatest synergistic effect is with clindamycin. In contrast, ampicillin shows no synergistic effect, the inhibition of the formation of MRSA biofilms being the same as for Ag-NPs alone (58%); for the others the inhibition ranges from 62-69%.

Iron atoms have 4 unpaired electrons in the 3d shell; Fe^2+^ ions have also 4 and Fe^3+^ have 5. Therefore, when they form crystals they have a strong magnetic moment, and can be ferromagnetic, antiferromagnetic or ferrimagnetic. Magnetic NPs, between 2 and 20 nm in diameter, display superparamagnetism 149. The two main forms of superparamagnetic iron oxide NPs (SPIONs) are magnetite (Fe_3_O_4_) and its oxidized form, maghemite (γ-Fe_2_O_3_). In the absence of an external magnetic field, their magnetic properties are not displayed. Magnetic NPs in solution can absorb the energy of an alternating magnetic field and convert it into heat 150. These properties make them ideal candidates as:

- vectors for drug delivery 151,
- contrast agents for magnetic resonance imaging (MRI) 152,
- platforms for theranostics (by combining drug delivery and MRI) 153,
- therapeutic agents for magnetic and optical hyperthermia in anti-cancer therapy 154,
- agents for regenerative medicine 155,
- magnetic biosensors 156.

Their toxicity is highly dependent on the coating, e.g. 10 nm IONPs coated with PEG are non-toxic while IONPs coated with polyethylenimine (PEI) exhibit dose-dependent lethal toxicity. The size of the IONPs is another parameter that affects toxicity, and biodegradation and clearance also depend on it. IONPs are usually distributed in the liver and spleen, with rather less in other organs such as lungs, heart and kidneys. Their main mechanism of bactericidal action is through the generation of ROS leading to cell death by necrosis or apoptosis 157**.** Hydroxyl radicals are generated inside lysosomes by the Fenton reaction of free iron, Fe^2+^ and hydrogen peroxide. α-Fe_2_O_3_ increases the growth of *P. aeruginosa* biofilms 158. This is because the smallest NPs release Fe^3+^ ions which interact with antimicrobial proteins, such as lactoferrin, believed to prevent colonization 159. Lactoferrin is an iron-binding glycoprotein that sequesters free iron. It can be bactericidal by binding lipopolysaccharide of bacteria walls and results in cell breakdown (lysis) by interacting with the oxidized iron part of the bacteria.

IONPs are used as contrast agents in MRI to visualize the sites of bacterial infections of several species, such as *S. aureus*, *H. pylori* and *Mycobacterium tuberculosis* 160. MRI can visualize anatomical abnormalities in 3D with high resolution. Superparamagnetic IONPs reduce the longitudinal and transverse relaxation times of surrounding protons.

#### 5.2.2 pH-Responsiveness

A pH-dependent liposomal NP, coated with a quaternary ammonium, chitosan (a QAC), was designed to attack *Porphyromonas gingivalis* biofilms 161. The environment of dental biofilms in humans has a low pH, around 4.5 or less, and the liposome NPs are soluble under both acidic and basic conditions thanks to the positive charges on chitosan, a strong polyelectrolyte. These charged species target the anionic matrix of some biofilms by strong electrostatic interactions 162. Under acidic conditions, the amines of the QAC are protonated, and act as pH-responsive domains, thus destabilizing the liposomal NP, which releases an encapsulated drug, doxycycline hydrochloride, effective against Gram-positive and Gram-negative bacteria.

Likewise, other pH-responsive PNPs have been designed for drug delivery in oral biofilms of *S. mutans*. pH-Responsive p(DMAEMA)-b-p(DMAEMA-co-BMA-co-PAA) block copolymer micelles are cationic, have a high affinity for dental biofilms, and encapsulate farnesol, an antibacterial drug 163. Another pH-responsive PNP, composed of poly(ethylene glycol)-block-poly(2-(((2-aminoethyl)carbamoyl)oxy)ethylmethacrylate) (PEG-b-PAECOEMA) encapsulating an antibacterial, chlorhexidine, eradicates biofilms formed by *S. mutans* 164*.*

Au-NPs functionalized by zwitterionic pH-sensitive ligands consisting of 11-mercapto-undecanoic acid (HS-C_10_-COOH) and (10-mercaptodecyl) trimethylammonium (HS-C_10_-NMe_3_) bromide are effective against MRSA biofilms 165. The zwitterionic compound adheres strongly to the surfaces of negatively charged (pH ∼5.5 vs. 7.4 in healthy tissue) bacteria in MRSA. Au-NPs aggregate in biofilms, but their photothermal properties make this an advantage. Under near-infrared (NIR) irradiation, MRSA biofilm is destroyed but surrounding healthy tissues are undamaged, with the dispersed Au-NPs having no photothermal effect. In the acidic environment of biofilms, a nanohybrid of SiO_2_-P_Ce6_-IL becomes positively charged and, by interacting with negatively charged EPS, creates holes in MRSA biofilms 166. This nanohybrid is also a photosensitizer, and in combination with the holes created within the biofilm, significantly improves photodynamic therapy (PDT) against MRSA.

#### 5.2.3 Matrix disruption

Certain polymer NPs containing drugs disrupt bacterial biofilm, but also act on planktonic bacteria 167. Polymer NPs break bacterial biofilms either by delivering therapeutic molecules or by modifying their surface with antibacterial components, like alkyl-pyrimidines or QACs. Cationic functions interacting with the anionic matrix of biofilms have bactericidal activity, by destructuring cell membranes 168. PLGA-coated NPs, functionalized with the enzyme DNase I, and encapsulating ciprofloxacin, have been designed for the eradication of *P. aeruginosa* biofilms. They penetrate and disrupt the matrix structure of the biofilms, locally releasing ciprofloxacin which then acts even on the bacteria most deeply buried in the biofilm. Based on the structure of antimicrobial peptides, synthetic semi-rigid polymers have been designed, with hydrophobic and cationic domains 169. A library of quaternary ammonium-poly(oxanorborneneimides) with different degrees of hydrophobicity shows excellent therapeutic indices as well as very low toxicity to red blood cells. These polymeric NPs (PNPs) penetrate and eradicate biofilms of *P. aeruginosa, En. cloacae* and *S. aureus*. Due to the highly cationic and hydrophobic nature of PNPs, it has been suggested that their activity arises from the disruption of bacterial cell membranes. Even after several treatments, the bacteria do not develop any resistance towards this nanomaterial.

Another example: under UV irradiation thin-film composite membranes (TFCs) infused with TiO_2_ NPs have a photocatalytic bactericidal effect on *E. coli* 170, by disrupting the bacterial membrane and inhibiting attachment to its surface.

### 5.3 Chemical effects

#### 5.3.1 Generation of ROS

Nanomaterial-based catalysts are heterogeneous materials mainly based on metallic NPs whose high specific surface area increases their catalytic activity. A catalytic NP (CAT-NP) with peroxidase-like activity has been designed to destroy dental *S. mutans* biofilms 171. It contains biocompatible Fe_3_O_4_, which has been developed to catalyze the decomposition of H_2_O_2_ at low pH, in order to generate free radicals *in situ*, which simultaneously degrade the biofilm matrix and rapidly decrease bacterial proliferation. Likewise, dextran-coated IONPs, termed nanozymes (Dex-NZM), display strong peroxidase-like catalytic activity at low pH 172. This nanocomposite targets biofilms with high specificity, penetrating the matrix of *S. mutans* biofilms, and locally killing bacteria by disruption of the structure. Ferumoxytol (Rienso® in Europe) is an intravenous treatment for iron deficiency. This superparamagnetic IONP disrupts intractable oral biofilms of *S. mutans* and prevents tooth decay in the same way 173. Ferumoxytol binds to the biofilm matrix and generates free radicals from H_2_O_2_, causing bacterial death by rupture of the cell membrane and degradation of the EPS matrix.

Ag-NPs capable of diffusing into the biofilm (diameter about 40 nm) completely inhibit the formation of *S. mutans* and *E. coli* biofilms by the generation of ROS 174. Very recent work shows that they inhibit the formation of biofilms (> 99%) of *P. aeruginosa*, *E. coli* and *S. aureus* 175 and the formation of MRSA biofilms by producing ROS. Also, they decrease the production of EPS of *K. pneumoniae* biofilms by 44-45% (dry weight) as well as inhibiting biofilm formation by 23-86% 176.

MgF_2_ NPs show antibiofilm activity against *S. aureus* and *E. coli* 177. They attach to the surface and penetrate into the biofilm, thus disrupting the structure by inducing bacterial membrane lipid peroxidation. ROS are produced and damage unsaturated acids in cell membranes, lipoproteins or other molecules. These NPs inhibit the formation of biofilm for three days, and at the end of this period, the formation of colonies of isolated bacteria resumes.

Nitric oxide (NO)-releasing silica NPs show antibiofilm effects against *P. aeruginosa*, *E. coli*, *S. aureus* and *S. epidermidis* 178. NO is a reactive free radical produced by neutrophils and macrophages to fight infection during inflammation. Due to its antimicrobial capacity, its small size and fast diffusion, NO destroys bacteria embedded in the matrix, and kills ≥ 99% of the bacteria in the biofilms, with the highest mortality for those of *P. aeruginosa* and *E. coli*. In addition to killing them, NO disperses bacteria in *P. aeruginosa* biofilms. These NPs are as toxic to fibroblasts as, or less toxic than, the antiseptics currently used, and they have advantages for healing wounds.

#### 5.3.2 Inhibition of QS

Silencing communication between the bacteria of the biofilms, QS, is a therapeutic strategy. Silicon dioxide NPs (Si-NPs) 179, functionalized with β-CD, emit luminosity proportional to the concentration of acylated homoserine lactones (AHLs) of QS of *Vibrio fischeri* biofilms. These bacteria use AHL as a means of coordinating their activities, but also for bioluminescence 180. β-CD binds non-specifically to AHL, reducing the luminosity; the bacteria are therefore no longer able to communicate and find themselves isolated.

A herbal metallic colloidal nano-formulation (Uh-Au@Nano-CF), containing Swarna (gold) NPs and polyphenols from a *Usnea longissima* extract (a medicinal lichen), has anti-QS properties 181. It degrades the structure of biofilms, as well as inhibiting their formation. *U. longissima* also contain usnic acid, which inhibits biofilm formation or eradicates preformed biofilms at higher concentration. There is a synergistic effect between the lichen and the biogenic Au-NPs.

Silver (Ag-NP), Selenium (Se-NP) and tellurium (Te-NP) NPs are active against *P. aeruginosa* biofilms 182. Se-NPs have very interesting antioxidant, anticarcinogenic and antimicrobial properties 183. Compounds based on tellurium are used in semiconductors, rechargeable batteries and in glasses, but they also have anti-inflammatory, antimicrobial and anticarcinogenic properties 184. Se-NPs inhibit 60-70% of *P. aeruginosa* biofilm formation, by disrupting QS signals so that the biofilm can no longer develop. However, they are less efficient in removing an already formed biofilm (only 15%). For Te-NPs, the efficiency is 80% for the inhibition of biofilm formation, but only 30% for the removal of preformed biofilm.

#### 5.3.3 Localized antibacterial action

Nanomaterials offer as well several possibilities for creating antibacterial coatings on implants or medical devices that allow for a localized antibacterial action and decrease risk of biofilm formation. A localized antibacterial action at the site of an implant or on the surface of the medical device can be effective in preventing the initial steps of biofilm formation. In the case of implants the greatest risk is at the moment of the operation when the body is opened. However, the risks remain until the body is closed. A localized antibacterial action can be achieved by encapsulating antibiotics or other active molecules on the surface of implant/device or by incorporating an antibacterial material. For example, nanoscale features on surfaces offer a means of reducing bacterial proliferation, as shown by surface deposition of Ti nanotubes 185. Incorporating graphene nanomaterials into a titania matrix increases their conductivity, enhancing transfer of extruded electrons from the bacterial cell membrane to the composite and subsequent electron enrichment at the Schottky-like interface, which in turn leads to bactericidal action 186. Nanostructured mesoporous films of titania can be used for on-top implant loading of antibiotics, which are then released at the implant site 186. Mesoporous titania films have been loaded with gentamicin. In physiological media there is an initial burst release of gentamicin followed by a prolonged release that lasts weeks. This slow release is explained by the interaction of OH groups from gentamicin with the walls of the titania pores. Such a release profile is highly appealing for bone implants where a high concentration of antibiotics is necessary during surgery, while a lower concentration is needed until tissue is regenerated 187.

Nanoscale polymer coatings have been extensively used to confer antibacterial properties on implants and medical devices. Polyelectrolyte multilayers fabricated by the Layer-by-Layer (LbL) technique deserve a special mention. This technique is based on the alternating assembly of oppositely charged polyelectrolytes by electrostatic interactions, and can be applied to the non-covalent modification of multiple substrates, including medical implants, provided that these are charged or can be charged. LbL assembly results in nanostructured layered films, with polymer layers a few nanometers thick, in which other charged molecules or NPs can be assembled or complexed 188. Very interesting strategies have been developed for the loading of LbL films with antibiotics 186, antibacterial peptides, and bactericidal NPs. Moskowitz *et al.* 189 proposed a LbL-based antibacterial coating, consisting of a tetra-layer unit containing gentamicin sulfate, polyacrylic acid and a synthetic poly(β-amino ester). The entire film comprised up to 200 tetra-layers, fabricated over 5 days by an automated procedure. It gave an initial burst release of gentamicin followed by slow release. Coatings with 100 tetra-layers had a bactericidal effect against *S. aureus*. In a similar fashion Escobar *et al.* fabricated LbL multilayers from complexes of polyacrylic acid, gentamicin and polylysine. The complexes formed in slightly acid solution hold large amounts of gentamicin, which is released after LbL assembly at neutral pH without compromising the stability of the film 188. LbL coatings can also restrict bacterial adhesion to surfaces. Moreover, the sequential assembly of building blocks allows the combination of antibiotics, peptides and NPs all in one, enhancing antibacterial action.

#### 5.3.4 Photothermal therapy and Photodynamic therapy

Photothermal therapy (PTT) and photodynamic therapy (PDT) have received considerable attention and are recognized as viable alternatives for treating biofilm infections 190. PTT increases the penetration of antibacterial agents into biofilm and decreases the progression of antibiotic resistance. Irradiation in the near infrared (NIR) reduces biofilm by ca. 90%, indicating the therapeutic efficacy of localized antimicrobial exposure and hyperthermia 191. However, the high irradiation doses and photosensitizer concentrations used to ablate biofilms by PTT and PDT may cause severe tissue damage and inflammation 192.

Combined therapy can improve the therapeutic efficacy and reduce side-effects in the treatment of bacterial biofilm infections 193.

New research is now oriented towards the use of smart designs, based on hydrogels, antibacterial drugs, plasmonic nanoplatforms, etc. Moreover, synergistic methods are sought as promising alternatives for treating bacterial infections and in fighting biofilm formation.

The achievements related to the applications of nanomaterials in biofilm inhibition are summarized in Table 4.

## 6. New developments

In contrast to conventional treatments which consist in simply administering a drug and waiting for it to take effect, in innovative approaches an NP, which may be associated with a drug, is stimulated by a magnetic field or laser irradiation in order to produce a local temperature increase, to release the drug and/or to mechanically disrupt the biofilm. Drug efficacy is notably enhanced by association with NPs stimulated in this way.

### 6.1 Laser irradiation

Hybrid metal-polymer NPs enable complete destruction of *S. aureus* and *P. aeruginosa* biofilms using laser-induced direct transfer (LIFT) 194. LIFT is a process that uses a pulsed laser beam as the driving force for depositing a thin layer of an organic or inorganic donor substrate onto an acceptor material with high spatial resolution. This transfer can be carried out in the solid or liquid phase. By LIFT *S. aureus* and *P. aeruginosa* biofilms, which act as receptors, were put into direct contact with a metal NP-polymer composite of a thin metallic film of silver, copper or gold on a polyethylene terephthalate substrate. The laser alone had no effect on the biofilms. In contrast, the silver and copper NPs completely destroyed them.

### 6.2 Magnetic disturbance

In a very recent article, IONPs (Fe_3_O_4_ and γ-Fe_2_O_3_) damage the matrix of MRSA biofilms when a magnetic field is applied 195. The magnetic field controls and concentrates the IONPs at a precise point. The highest antibiofilm activity is for 11 nm IONPs (as against 8 nm and 70 nm), and the two applied magnetic fields, AC and DC, facilitate biofilm eradication more than direct contact. IONPs fail to kill the planktonic MRSA bacteria; they only act upon the physical disruption of the biofilm, thus releasing the biofilm from the surface. A rotating DC magnetic field disperses biofilms the best. A low rotation rate allows the IONPs prolonged contact with the matrix, generating significant shearing forces within the biofilm. The magnetic field and the IONPs therefore act as "shield breakers". A nanocarrier, polymersome, encapsulating IONPS and methicillin, penetrates *S. epidermidis* biofilms to a depth of 20 µm in a magnetic field. The IONPs partially destructure the biofilm, improving the contact of the antibiotic which, by a synergistic effect, completely destroys the sessile community 196.

The application of an AC magnetic field to IONPs to cause heating is known as magnetic hyperthermia. Local heating by IONPs is much larger than that of the medium, and the associated “hot-spots” are responsible for the detachment of the biofilm. Mild magnetic NP hyperthermia increases the sensitivity of *S. aureus* biofilms to conventional antibiotics 197.

Block copolymer poly((oligo(ethyleneglycol)methyl ether acrylate)-*block*-poly(monoacryloxyethyl phosphate))-coated IONPs disrupt the structure of *P. aeruginosa* biofilms by magnetic hyperthermia, 198. The increase in the local temperature, from 23 °C to 40 °C, makes it possible to detach and disperse the biofilms. The temperature-sensitive regulatory messenger, c-di-GMP, is inactivated by hyperthermia 199. This in turn activates the LapG protein which cleaves adhesins to trigger the detachment of the biofilm. This hybrid nanomaterial also allows improvement of antibiofilm therapy of *P. aeruginosa*, by combining it with gentamicin, usually used against this bacterium. It has been suggested that IONPs are able to create artificial channels, thereby improving the transport of antibiotics within the biofilm 200. The idea is that in a magnetic field the NPs sink into the biofilm and force the creation of channels due to their passage from the surface to a point closest to the magnet. In a magnetic field, IONPs make *S. aureus* biofilms 4-6 times more sensitive to gentamicin.

## 7. Conclusions

The current state-of-the-art indicates that enhanced antimicrobial tolerance is a general trait of biofilms and is the result of different specific factors, which depend on the species of bacterium involved, the environment of the biofilm and the choice of the antimicrobial agent. There is still no strategy that allows biofilms to be consistently and efficiently eradicated: effective, easy and safe-to-use, novel antimicrobial agents are urgently required. The extracellular matrix and specific physiology (including slow-growth and membrane rigidification) play important roles in the low activity of antimicrobials on biofilms. This is why it is urgent to deepen current knowledge on biofilm formation and degradation, and on the interaction of materials with biofilms, in order to create strategies to fight them. Development of novel materials and approaches to cope with the risks of bacterial infections and pandemics in the long term, that can be applied for a large number of scenarios, regardless of the bacterial strain, is fundamentally important. At the same time, we must think about individual cases, aiming to fight specific aggressive bacterial strains and direct treatment by a more personalized medical approach. Coping with these two issues is where the big scientific challenges in the fight against biofilms and bacterial infections lie.

## Figures and Tables

**Table 1:** Diseases and infections associated with biofilms

Location	Bacteria	Consequences	Refs
**Oral cavity**	*Streptococcus mutans, Aggregatibacter* (formerly *Actinobacillus*) *actinomycetemcomitans*, *P. gingivalis*, *P. intermedia*, *E. corrodens*, and oral spirochetes	Development of periodontal infections near the gums, with emergence of highly pathogenic biofilms that induce acute inflammatory reaction, leading to breakdown of periodontal tissues and possibly to loosening of teeth. Also, the structure of the tongue promotes the formation of a unique and complex bacterial biofilm, in which odor-producing periodontal pathogens are frequently found, resulting in halitosis.	33,34
Otitis media	*Streptococcus pneumoniae*, *Haemophilus influenzae*, and *Moraxella catarrhalis*	Complex set of infectious and inflammatory conditions affecting middle ear	35
Musculoskeletal system	*S. aureus*, coagulase-negative staphylococci, *Enterococcus spp*. and *Streptococcus spp.*	Bacteria aggregate on dead bones (sequestering), or on implants leading to biofilm infections	36
Necrotizing fasciitis	Group A streptococci, but other bacteria can be the cause (especially those that grow in water, such as *Vibrio spp.*)	Necrotizing fasciitis: serious infection that causes death of skin tissues and those underneath (subcutaneous, muscles). Causes tissue necrosis, often requires amputations or major surgical operations. Can also lead to heart disease, systolic shock (blood pressures <90 mm Hg) and clostridial infections	37
Cystic fibrosis	*P. aeruginosa* and* S. aureus*	Also called mucoviscidosis, affecting the lungs, kidneys, and digestive system. If severe form develops, lung transplant may be only solution. Build-up of mucus in lungs causes lung infections. Most common symptoms are: persistent cough with thick discharge from mucus, inflamed nasal passages, shortness of breath, wheezing and salty-tasting skin	5

**Table 2:** Biofilms on medical equipment

Medical equipment	Location	Bacteria	Disease	Refs
Catheters	Intravascular catheters (IVCs), as well as central venous catheters (CVCs)	Coagulase-negative Staphylococci, Gram-negative rods, *Staphylococcus aureus* (*S. aureus*), and *Candida albicans*	Catheter-related bloodstream infection (CRBSI)	39
	In-dwelling catheters	*E. coli*, *Enterococci*, *P. aeruginosa*, *Klebsiella pneumonia*, *Candida albicans*, *Enterobacter*, *P. mirabilis* and coagulase-negative *Staphylococci*	Catheter-associated urinary tract infection leading to cystitis, pyelonephritis, Gram-negative bacteremia, prostatitis, epididymitis, endocarditis, vertebral osteomyelitis, septic arthritis, endophthalmitis and meningitis	40
Heart valves	Mechanical heart valves, on surrounding tissues or on reconstructed native heart valves	*S. epidermidis*, *S. aureus*, *Streptococcus spp*., Gram-negative bacilli, diphtheroids, enterococci, and *Candida spp*	Prosthetic valve endocarditis, infection of peripheral tissues	41
Orthopedic prostheses	Joint or bone replacement	Gram-positive cocci: *S. aureus*, coagulase-negative staphylococci and enterococci	Infections associated with prostheses, as well as dissemination to other sites	42
Implants	Endotracheal and tympanostomy tubes, orthopaedic and breast implants, contact lenses, intrauterine devices (IUDs), sutures and vascular grafts	*S. aureus* (generally found on metallic implants and in acute infections), coagulase-negative staphylococci (*S. epidermis*), beta-hemolytic streptococci, and aerobic Gram-negative rods	Acute local inflammatory reactions, that can persist as chronic inflammation, destruction of bone tissue (osteolysis), as well as mechanical loosening (aseptic)	38,43

**Table 3:** Bactericidal efficiency of Sidr and Manuka honeys against MRSA (Methicillin-resistant *S. aureus*), MSSA (Methicillin-sensitive *S. aureus*) and *P. aeruginosa* biofilms

	Sidr honey	Manuka honey
MSSA biofilm	63%	82%
MRSA biofilm	73%	63%
*P. aeruginosa* biofilm	91%	91%

**Table 4:** Application of nanomaterials in biofilm inhibition

Nanomaterials	Mode of action	Bacteria	Biofilm impact	Refs
Drug delivery:	Drug carrier			
Liposomes	hydrophilic, lipophilic, amphiphilic	*S. aureus, P. gingivalis*	Slow down, growth inhibition	105-108
SLNs	Prolonged release : hydrophilic, lipophilic,	*S. aureus*	Growth inhibition	109-112
QSIs	Anti-agglomeration, anti-aggregation	*P. aeruginosa, V. fischeri*	Eradication, growth inhibition	113, 177-178
PNPs	hydrophilic, hydrophobic	*E. coli, S. mutans, S. aureus, P. aeruginosa, En. cloacae*	Matrix disruption, eradication, growth inhibition	114, 164-166
Dendrimers	hydrophilic, hydrophobic	*S. aureus, E. coli*	Antimicrobial	115-117
Cyclodextrins	hydrophobic	*C. albicans, S. aureus, P. aeruginosa, E. faecalis, P. vulgaris*	Adhesion inhibition, eradication	121-123
Hydrogels	Bacteriophage, hydrophilic, hydrophobic	*P. aeruginosa, S. aureus, MRSA, Acinetobacter baumannii*	Biofilm eradication, wound healing	127-130
Stimuli responsive NPs:	Intrinsic properties:			
SPIONs	Magnetic disturbance, ROS generation, thermal therapy, drug delivery	*P. aeruginosa, H. pylori, M. tuberculosis, S. aureus, S. mutans*	Oxidative stress, cell lysis, colonization prevention	153-156, 133-135, 170
Ag-NPs	ROS generation, antibacterial, drug carrier	*P. aeruginosa, E. coli, S. aureus, K. pneumoniae, S. flexneri, S. mutans*	Oxidative stress, inhibition, genetic mutation, structural alteration	128-131, 135-140
Au-NPs	Thermal and photodynamic therapies, photosensitizer	*S. aureus, P. aeruginosa, E. coli, C. albicans*	Matrix disruption, growth prevention,	132-134, 167-169
Other inorganic NPs	Photocatalysis, ROS generation, antimicrobial, antioxidant	*E. coli, S. aureus, P. aeruginosa, S. epidermidis*	Matrix disruption, growth inhibition	132, 173-179
